# Transcriptome Analysis Reveals Calcium-Induced Defense Response in Barley Against *Magnaporthe oryzae*

**DOI:** 10.3390/ijms27156544

**Published:** 2026-07-23

**Authors:** Juan Zhao, Xiang Li, Feng Zhang, Chengming Zhang, Rui Zeng, Hongling Liu, Songqing Liu

**Affiliations:** Sichuan Provincial Key Laboratory for Development and Utilization of Characteristic Horticultural Biological Resources, College of Chemistry and Life Sciences, Chengdu Normal University, Chengdu 611130, China; 091091@cdnu.edu.cn (J.Z.);

**Keywords:** barley, calcium ion, disease resistance, transcriptome

## Abstract

In this study, we found that exogenous 50 mM calcium chloride treatment reduced the number of barley leaf lesions per plant by 39%. To investigate the molecular basis for this increased resistance, we used *Magnaporthe oryzae* spores to infect barley and collected the leaves for analysis. Four groups were set up: control, pathogen inoculation, 50 mM CaCl_2_ treatment, and 50 mM CaCl_2_ treatment plus pathogen inoculation for transcriptomics. Our results showed that both calcium treatment and pathogen inoculation altered the expression of many genes, which were enriched in linoleic acid metabolism, cutin, suberine and wax biosynthesis, flavonoid biosynthesis, phenylpropanoid metabolism, and plant–pathogen interaction pathways. Notably, qRT-PCR analysis revealed that the expression levels of disease-resistance-related genes *THN3*, *RGA4*, and *FAR4* were significantly reduced in the CaCl_2_-treated and pathogen-inoculated group compared with the control. Our findings demonstrate that exogenous calcium modulates gene expression and regulatory networks associated with barley disease resistance.

## 1. Introduction

Barley (*Hordeum vulgare* L.) is one of the most important cereal crops cultivated worldwide [1,2,3]. During growth, barley is often infected by various fungal diseases, which severely affect its yield and quality [4,5]. *Magnaporthe oryzae*, a major fungal pathogen of rice, can also infect barley [6,7]. In plant pathology research, the interaction between *M. oryzae* and barley serves as an important model system for studying host–pathogen interactions, non-host resistance mechanisms, and effector biology [8,9]. In addition, this model has also contributed to the development of various advanced detection techniques for disease management [10,11,12].

The latest research reveals that the resistance of barley is specifically against *M. oryzae*, and this specificity depends not only on the genetic background of the pathogen but is also profoundly influenced by the regulation of the immune receptor network in barley [13]. Regarding the interaction between barley and *M. oryzae*, it has been demonstrated that the barley immune receptor *MLA3* confers resistance to blast disease by directly recognizing the effector *Pwl2* of *M. oryzae*. In addition, the *Rar1* gene is related to the immune signaling pathway in barley, and loss of *Rar1* leads to increased susceptibility of barley to the blast fungus [14]. Notably, abscisic acid increases susceptibility to blast disease by suppressing penetration resistance in barley [15]. In the plant and fungus interaction, the effectors *MoHEG13* and *MoHEG16* interfere with the host infection process in barley [16]. Proteomic analysis further revealed that, upon *M. oryzae* infection, barley initiates a specific apoplastic protein defense network at the early stage of infection to restrict hyphal expansion [17]. Apart from these findings, little research has been conducted on blast resistance in barley.

Calcium (Ca) is an essential nutrient for plants and plays important physiological roles in plant growth and development [18]. Existing studies have focused on the role of exogenous calcium in alleviating abiotic stresses such as drought, flooding, salinity, high temperature, low temperature, heavy metals, and acid rain, while studies on biotic stress are relatively limited [19,20,21]. It has been shown that pretreatment with calcium chloride (CaCl_2_) can effectively enhance drought tolerance in different barley varieties [22]. Notably, the beneficial effect of this treatment interacts significantly with the genetic background of the variety. In addition, calcium promotes the antioxidant enzyme activities and improves the scavenging efficiency of reactive oxygen species, thereby alleviating stress-induced damage such as aluminum toxicity [23,24].

Calcium also plays a central role in the plant immune system. Exogenous calcium treatment enhances the resistance of peanut to *Phoma arachidicola* by improving cell membrane stability and regulating ROS metabolism [25]. In winter jujube fruit, phenyllactic acid strengthens resistance to *Alternaria alternata* through modulation of Ca^2+^ signaling and the MAPK cascade [26]. Calcium also restricts pathogen spread by inducing phytoalexin biosynthesis and cell wall reinforcement [27]. In *Arabidopsis*, the loss of proton/calcium exchanger 1 results in activation of defense responses and accelerated senescence, indicating that calcium homeostasis is crucial for immune regulation [26]. Calcium chloride can serve as an effective inducer to enhance barley disease resistance, and its superior ability to promote phenolic compound accumulation provides an important approach for controlling spot blotch disease [28].

Although numerous studies have demonstrated that exogenous calcium treatment can enhance stress tolerance in barley, the specific genes involved in disease resistance regulation in barley remain largely unknown. To address this, we established multiple treatment groups, including calcium-treated versus untreated, and pathogen-inoculated versus non-inoculated combinations and screened candidate disease-resistance-related genes using comparative analysis of transcriptomics. These results not only provide candidate target sites for calcium-mediated disease resistance in barley but also offer a theoretical basis for future barley resistance breeding and lay a foundation for green disease management strategies for calcium fertilizers.

## 2. Results and Discussion

### 2.1. Treatment with 50 mM CaCl_2_ Enhances Disease Resistance in Barley

**Figure 1:**
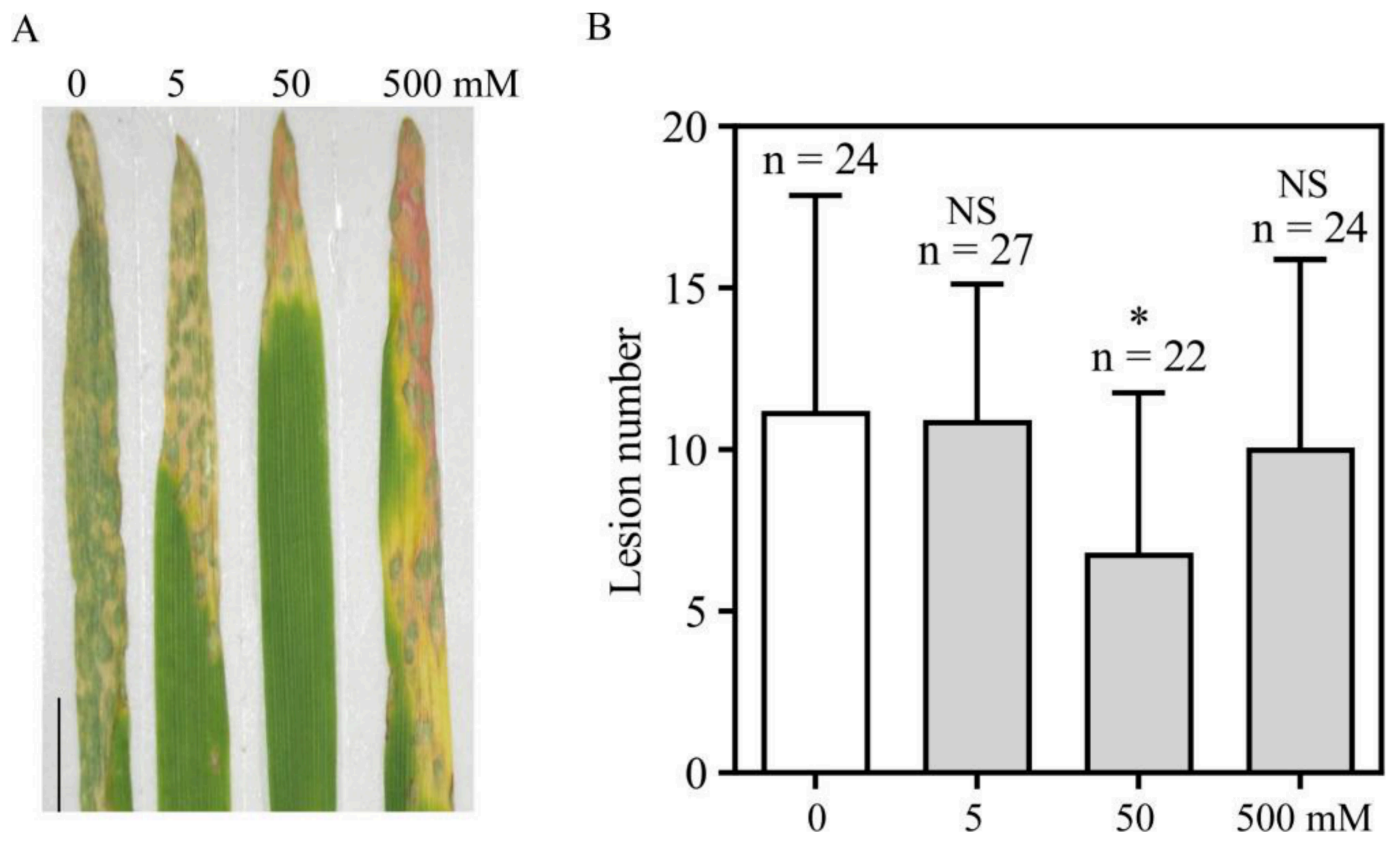
Enhanced barley resistance to rice blast when treated with 50 mM calcium solution. (**A**) Lesion development on barley leaves was observed after 5 days of inoculation. Bar, 1 cm. (**B**) Statistical analysis of lesion numbers per barley seedings; 0, 50, and 500 mM represent the barley seedings inoculated with the same volume of water and 50 and 500 mM CaCl_2_ solutions during planting, respectively. The number of lesions on the 50 and 500 mM calcium-treated barley seedings were compared to the 0 mM group and data were analyzed using *t*-tests. “n” indicates the number of barley seedings, “NS” indicates no significant differences, and asterisks indicate significant differences (*p* < 0.05).

To investigate whether CaCl_2_ treatment improves barley resistance to pathogens, barley seeds were soaked in the same volume of 0, 50, and 500 mM CaCl_2_ solutions for one day, then sown in soil. On the third day, 20 mL of 0, 50, and 500 mM CaCl_2_ solutions were applied to seedlings. The seedlings reached the one-leaf stage on the fifth day, then sprayed with the pathogen conidial suspension for inoculation. The number of lesions on barley seedlings was observed and calculated five days post-inoculation. Our results showed that the number of lesions on barley leaves which were treated with 50 mM CaCl_2_ solution exhibited a significant 39% reduction, while no significant differences were analysis with other CaCl_2_ concentrations. These results indicate that the 50 mM CaCl_2_ treatment significantly enhances disease resistance in barley (Figure 1).

### 2.2. Functional Analysis of Differentially Expressed Genes

To explore the molecular mechanisms underlying the enhanced disease resistance, we employed transcriptome analysis of the barley leaves with or without lesions, which were assigned to four groups: CK (blank, CaCl_2_ replaced by water), CK_P (water then pathogen), Ca (50 mM CaCl_2_ only), and Ca_P (50 mM CaCl_2_ then pathogen), each with three biological replicates (1/2/3). Leaf samples were collected to extract total RNA for transcriptome sequencing. Approximately 46 million clean reads and 7 Gb of clean bases were generated from each RNA-seq library. The average Q20 and Q30 values and GC content of each sample are 98%, 95%, and 55%, respectively. The total mapped reads of the CK, Ca, CK_P and Ca_P groups mapped to the *Hordeum vulgare* reference genome are 93%, 93%, 48%, and 62%, respectively (Supplementary Table S1). In barley–*M. oryzae* dual RNA-seq research, mapping to the barley genome was 48% (severe) vs. 62% (mild), mainly from fungal biomass increase after necrotrophy diluting host reads, not RNA degradation; host cell death and fungal RNases also lowered mapping [29,30]. Our results also indicate that calcium-treated significant decreased the fungal biomass in barley seedlings.

**Figure 2:**
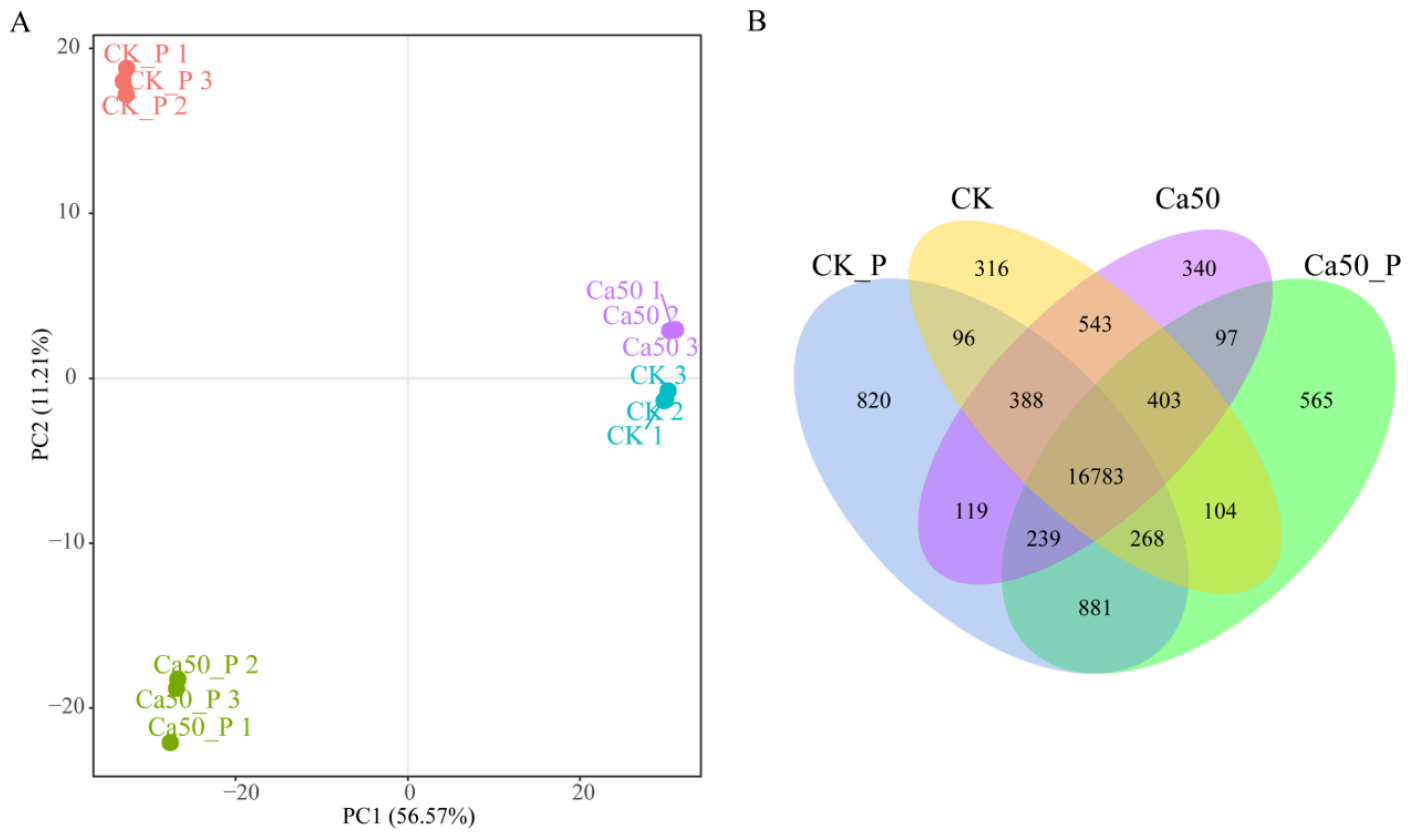
Functional analysis of expressed genes. (**A**) Principal component analysis plot. The x-axis represents the first principal component, and the y-axis represents the second principal component. (**B**) Venn diagram of co-expression. The number of uniquely expressed genes per group are shown, and intersections represent genes co-expressed across multiple groups.

Principal component analysis (PCA) was performed to assess intergroup differences and intragroup sample reproducibility. PCA uses linear algebra to reduce the dimensionality of thousands of gene variables and extract principal components. PCA was conducted on the gene expression of fragments per kilobase of exon model per million mapped fragments (FPKM) values of all samples. Our results showed that samples within each group exhibited good clustering, which is indicative of reproducible experimental conditions (Figure 2A). Samples from different groups were relatively dispersed; the Ca50 and CK groups were close to each other, whereas after inoculation, the CK_P and Ca50_P groups were more separated. These findings suggest that exogenous calcium treatment alone has little effect on the barley transcriptome, but the transcriptional response upon pathogen inoculation is substantially altered.

A co-expression Venn diagram showed substantial overlap of genes among groups. Specifically, 18,901 genes were detected in the CK group, 18,912 in the Ca group, 19,594 in the CK_P group, and 19,340 in the Ca_P group. Among these, the numbers of non-overlapping (unique) genes were 316 in CK, 820 in CK_P, 340 in Ca50, and 565 in Ca_P, indicating that exogenous calcium may confer enhanced disease resistance in barley through the modulation of specific genes (Figure 2B).

By comparing barley with and without calcium treatment, as well as before and after inoculation under calcium treatment, significantly differentially expressed genes (DEGs) were identified (padj ≤ 0.05, |log2FoldChange| ≥ 1.0). In the comparison between calcium-treated and non-treated samples, a total of 1121 DEGs were found, including 553 upregulated genes (49.3%) and 568 downregulated genes (50.7%). In the comparison with calcium treatment between inoculated and non-inoculated samples, a total of 1353 DEGs were identified, including 577 upregulated genes (42.6%) and 776 downregulated genes (57.4%) (Table 1). Additionally, we also compared barley inoculated with and without the pathogen; a total of 6542 DEGs were found, including 2835 upregulated genes (43.3%) and 3707 downregulated genes (56.7%). In the comparison with and without pathogen inoculation on calcium-treated barley, a total of 6572 DEGs were found, including 2900 upregulated genes (44.1%) and 3672 downregulated genes (55.9%) (Table 1).

Calcium treatment substantially reduced the transcriptional response of barley to *M. oryzae* infection, with DEGs decreasing from 6542 (without calcium) to 1353 (with calcium). Our results suggest that calcium may play a protective role by attenuating host reprogramming or limiting fungal proliferation.

**Figure 3:**
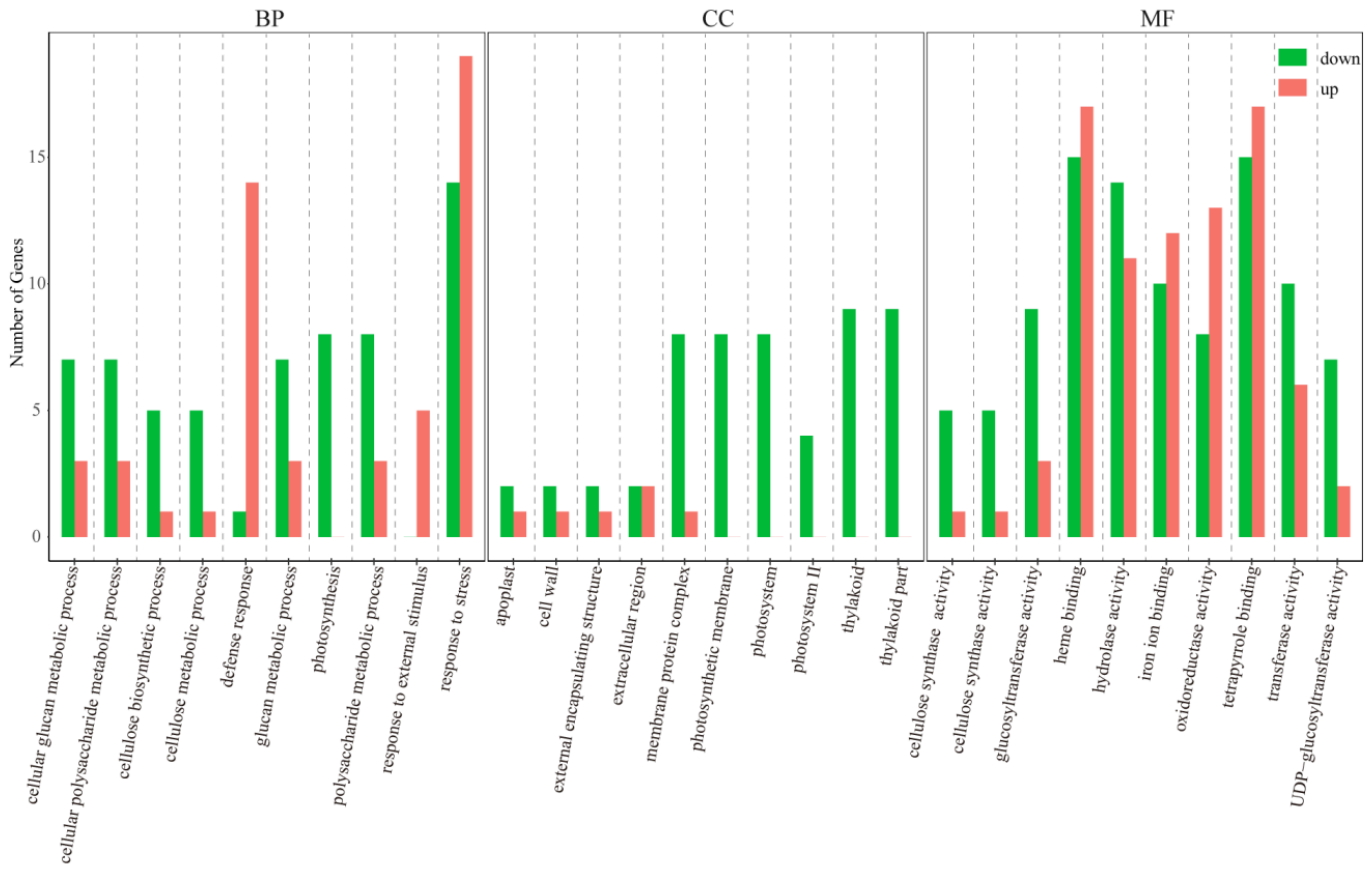
GO enrichment of CK VS Ca50. X-axis represents GO enrichment terms and Y-axis represents count of up-/downregulated differently expressed genes (DEGs) annotated. Green indicates downregulated DEGs and orange indicates upregulated DEGs. BP represents biological processes, CC represents cellular components, and MF represents molecular functions.

Gene Ontology (GO) enrichment analysis of the DEGs was performed to investigate the specific biological processes (BPs), cellular components (CCs), and molecular functions (MFs) affected by the enhanced resistance following calcium treatment. Select the top 30 terms with the lowest adjusted *p*-values from the GO enrichment results and visualize them in a bar chart. In the comparison between calcium-treated and non-treated barley, 33 DEGs were significantly enriched in BP terms related to stress response, comprising 19 upregulated and 14 downregulated genes. For MF, 32 DEGs were significantly enriched in hormone-binding molecular functions, including 17 upregulated and 15 downregulated genes (Supplementary Table S2). For CC, nine DEGs were significantly enriched in thylakoid components, all of which were downregulated (Figure 3).

**Figure 4:**
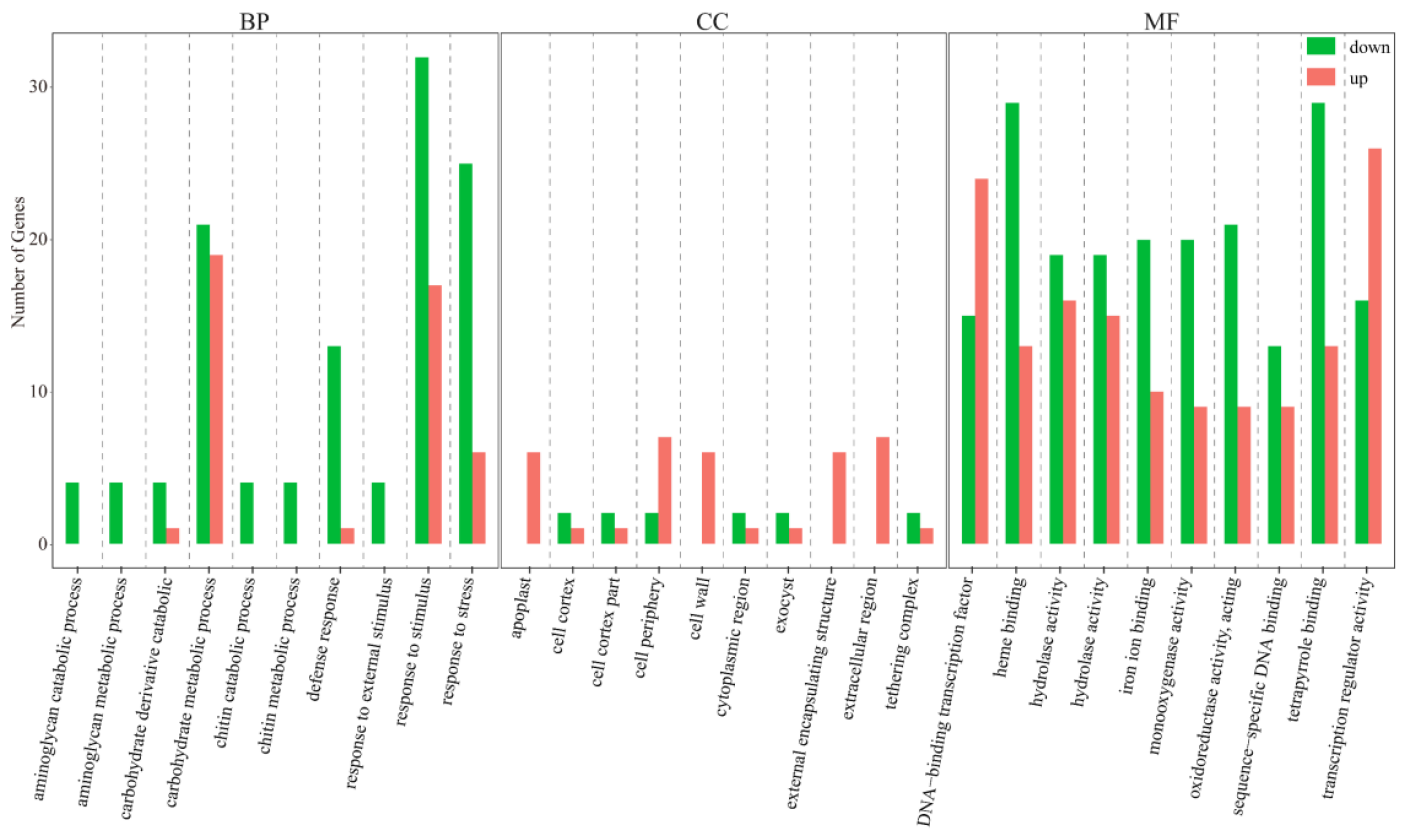
GO enrichment of CK_P VS Ca50_P. X-axis represents GO enrichment terms and Y-axis represents count of up-/downregulated differently expressed genes (DEGs) annotated. Green indicates downregulated DEGs and orange indicates upregulated DEGs. BP represents biological processes, CC represents cellular components, and MF represents molecular functions.

Analysis of DEGs in calcium-treated barley after inoculation revealed that 49 DEGs were significantly enriched in BP terms related to stimulus response, consisting of 17 upregulated and 32 downregulated genes. For MFs, 42 DEGs were significantly enriched in hormone-binding functions, including 13 upregulated and 29 downregulated genes (Supplementary Table S3). For CCs, nine DEGs were significantly enriched in the cell periphery, with seven upregulated and two downregulated genes (Figure 4).

**Figure 5:**
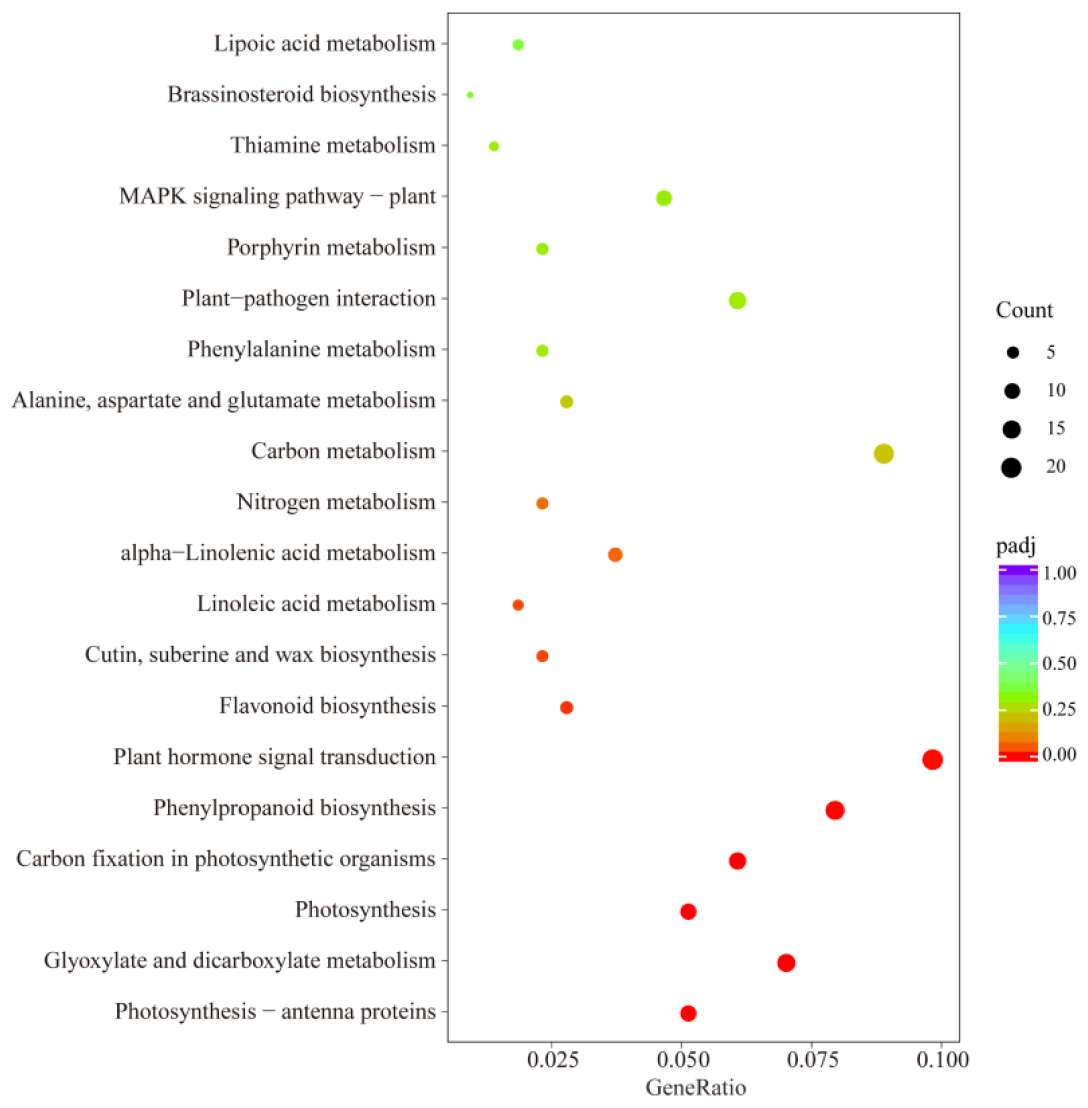
KEGG enrichment of CK VS Ca50. X-axis: ratio of GO-annotated DEGs to total DEGs; Y-axis: GO terms; dot size: number of annotated DEGs; color (red to purple): enrichment significance.

Based on the Kyoto Encyclopedia of Genes and Genomes (KEGG) enrichment results, the 20 most significant KEGG pathways were selected and visualized using a scatter plot. KEGG enrichment analysis of the DEGs showed that in the comparison between calcium-treated and non-treated barley, the pathways with the highest number of DEGs were plant hormone signal transduction and carbon metabolism; however, the most significantly enriched pathways were linoleic acid metabolism, cutin, suberine and wax biosynthesis, and flavonoid biosynthesis (Supplementary Table S4) (Figure 5).

**Figure 6:**
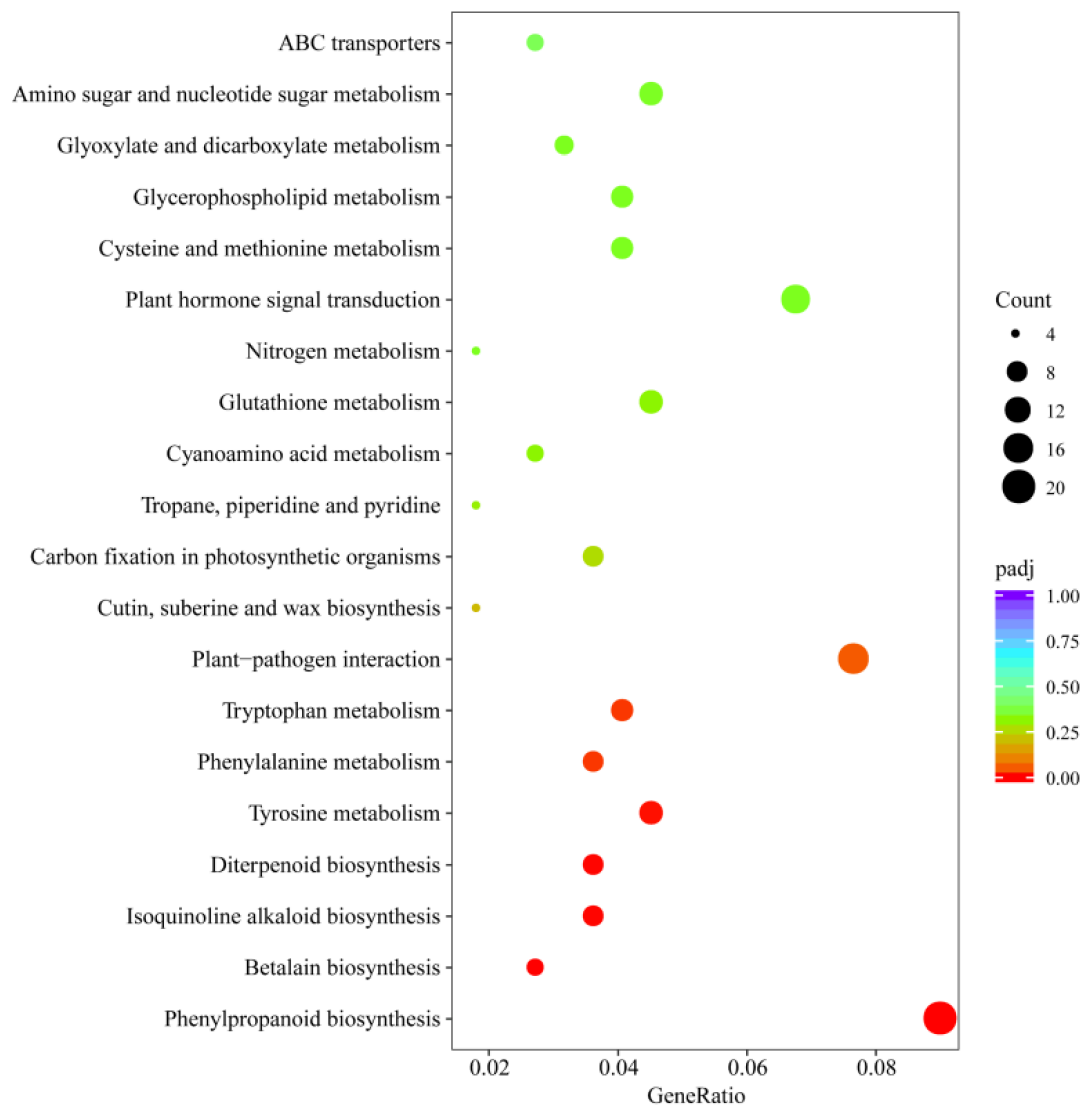
KEGG enrichment of CK_P VS Ca50_P. X-axis: ratio of GO-annotated DEGs to total DEGs; Y-axis: GO terms; dot size: number of annotated DEGs; color (red to purple): enrichment significance.

In calcium-treated barley after inoculation, the pathways with the most DEGs were phenylpropanoid metabolism and plant–pathogen interaction, whereas the most significantly enriched pathways were ABC transporters and amino sugar and nucleotide sugar metabolism (Supplementary Table S5) (Figure 6).

### 2.3. Screening of Calcium-Induced Disease Resistance Regulatory Genes

**Figure 7:**
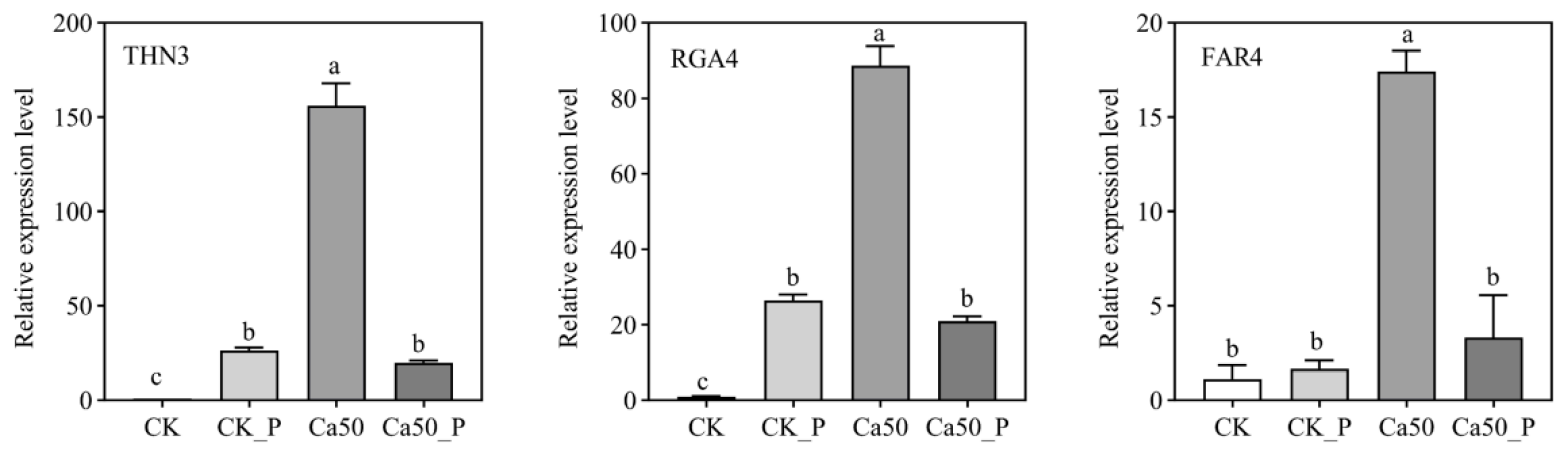
The expression levels differentially of expressed genes were detected by qRT-PCR. Means and standard deviations were calculated from three independent replicates. Data were analyzed using ANOVA analysis. Bars with different letters (a, b, c) represent significant differences at *p* < 0.05.

To identify key genes involved in exogenous calcium-induced disease resistance regulation, differentially expressed genes from the transcriptome were screened and validated. In this study, we aimed to identify calcium-regulated resistance genes. From our DEGs, we selected highly expressed genes with known roles in plant defense. Through screening, we identified HORVU_MOREX_r3_6HG0538430 (*THN3*), HORVU_MOREX_r3_1HG0001020 (*RGA4*), and HORVU_MOREX_r3_4HG0414900 (*FAR4*) as candidates. Relative to inoculated controls without calcium, these gene expressions were significantly upregulated after calcium treatment. Therefore, we performed qRT PCR assays to detect and verify the gene expression in the transcriptome using the 2^−ΔΔCt^ method with three technical replicates per group, and reference genes (*HvEF1a*) were validated with stable Ct values (SD < 0.5). Our results displayed that the trend of gene expression was consistent with the transcriptome data (Supplementary Table S6). *THN3* encodes a thionin in barley and is involved in defending against pathogen invasion. Following calcium treatment, *THN3* expression was significantly upregulated by 156-fold. After inoculation, *THN3* expression increased 26-fold under non-calcium treatment compared to CK, and 19-fold under calcium treatment. *RGA4* is homologous to the potato disease resistance gene *RGA4*. Its expression was significantly upregulated by 88-fold after calcium treatment. Upon inoculation, *RGA4* was upregulated in both groups: 26-fold under non-calcium treatment and 21-fold under calcium treatment. *FAR4* encodes a fatty acyl reductase homologous to that in *Arabidopsis*, participating in the formation of the extracellular lipid protective barrier. Calcium treatment resulted in a 17-fold increase in its expression. After inoculation, *FAR4* expression showed no significant increase relative to CK in either the non-calcium or calcium treatment groups (Figure 7). These results indicate that calcium treatment activates disease resistance genes (*THN3*, *RGA4*) and a physical barrier synthesis gene (*FAR4*), suggesting potential application value for enhancing crop immunity through calcium fertilization or manipulation of calcium signaling pathways.

## 3. Materials and Methods

### 3.1. Strains

The *Magnaporthe oryzae* strain P131 was cultured on oatmeal tomato agar plates and incubated in a constant-temperature light incubator at 28 °C with a 16 h light/8 h dark photoperiod [31].

### 3.2. Calcium Treatment

Healthy, plump barley (Fumai No. 2) seeds were selected for planting in soil; 40 mL of 5, 50, and 500 mM CaCl_2_ (Chron chemicals, Chengdu, China) solutions were used to soak seeds for one day under conditions of 28 °C, 65% humidity, and a 16 h light/8 h dark photoperiod, then planted in soil with 25 seeds per pot [32]. Subsequently, the seeds were sown in pots, and 20 mL of the corresponding CaCl_2_ solution was applied to the soil after three days of sowing. On the fifth day after sowing, the plants had grown to the one-leaf stage and their leaves had fully unfurled; the barley was inoculated for infection assays. Simultaneously, non-inoculated controls were established using the same CaCl_2_ treatment concentrations.

### 3.3. Infection Assays

A spore suspension was prepared using a 0.025% Tween-20 (Biosharp, Beijing, China) solution, and the spore concentration was adjusted to 10^5^ spores/mL [33]. Each cultivation box was sprayed with 5 mL of the spore suspension. After inoculation, the plants were kept in darkness under conditions of 28 °C and 80% humidity for 1 day. The number of leaf lesions was counted 5 days after inoculation. The number of lesions on diseased leaves was counted on a per-plant basis. Leaf images were captured using a digital camera, and the lesion numbers were analyzed using a *t*-test with GraphPad Prism 10 software.

### 3.4. Transcriptome Analysis

Diseased leaves were collected, and total RNA was extracted using the RNAprep Pure Plant Kit (TianGen, Beijing, China). RNA quality and integrity were assessed using an Agilent 2100 Bioanalyzer (Agilent Technologies, CA, USA) [34,35]. The extracted total RNA was used as sequencing samples, and three biological replicates were sequenced. Library construction was performed by mRNA enrichment, reverse transcription to synthesize double-stranded cDNA, end-repair, purification, and other steps. After quality verification, libraries were pooled and sequenced on an Illumina platform using sequencing-by-synthesis. Data quality control was conducted by removing adapter-containing, N-containing, and low-quality reads to obtain clean data. The reference genome and annotation files were downloaded (https://plants.ensembl.org/Hordeum_vulgare/Info/Index?db=core (accessed on 21 July 2024)), and the genome assembly represented here corresponds to the INSDC Assembly ID GCA_904849725.1. HISAT2 was used to build an index and align clean reads to the genome [35]. FeatureCounts was used to count sequencing reads, and FPKM values were calculated to quantify gene expression levels [36]. Subsequently, DESeq2 was used for differential expression analysis, with significantly differentially expressed genes identified using an adjusted *p*-value ≤ 0.05 as the criterion. To dissect the functions of differentially expressed genes, GO and KEGG pathway enrichment analyses were performed using the cluster Profiler software (version 3.8.1).

### 3.5. Quantitative Real-Time PCR (qRT-PCR)

Total RNA was reverse-transcribed into total cDNA using the Evo M-MLV RT Mix Kit with gDNA Clean for qPCR Ver.2 (Accurate biology, Changsha, China) according to the manufacturer’s instructions. The reverse transcription reaction (20 μL) was prepared on ice, incubated at 37 °C for 15 min and then at 85 °C for 5 s, and immediately cooled on ice. Real-time RT-PCR analysis of the differentially expressed genes identified in the transcriptome was performed using the SYBR Green Pro Taq HS PreMix Type III qPCR Kit (Accurate Biology) on a QuantStudio 6 Flex real-time PCR system (Thermo Fisher, Waltham, MA, USA) (primers listed in Table 2). Each 20 μL PCR reaction contained 10 μL of 2× SYBR Green mix, 0.4 μL of each forward and reverse primer (10 μM), 1 μL of cDNA template, and nuclease-free water to the final volume. The thermal cycling program consisted of an initial denaturation at 95 °C for 30 s; 40 cycles of 95 °C for 5 s and 60 °C for 30 s; followed by a melting curve stage of 95 °C for 15 s, 60 °C for 60 s, and a ramp to 95 °C at 0.05 °C/s. Three technical replicates were analyzed. *HvEF1a* was used as an internal reference, and the results were calculated using the 2^−ΔΔCT^ method [37]. Statistical comparisons were performed using ANOVA for multiple groups in GraphPad Prism 10 software, and *p* < 0.05 was considered statistically significant.

## 4. Conclusions

In this study, we demonstrate that exogenous calcium treatment enhances barley resistance to *M. oryzae*, resulting in a 39% reduction in lesion number (Figure 1). Transcriptomic analysis further revealed that calcium treatment activates a series of physiological and metabolic pathways associated with defense and stress responses. Therefore, we propose that calcium treatment is a defense enhancer, establishing a poised state that enables barley to mount a more rapid and robust response upon pathogen recognition.

We revealed that pathogen infection following calcium treatment activates the pathogen–host interaction pathway. This indicates that the calcium-induced resistance in barley is achieved through activation of the PTI (PAMP-Triggered Immunity) pathway [38]. After calcium treatment, PTI-related genes in barley were already enriched, and this enrichment further increased upon pathogen inoculation (Figure 5 and Figure 6). Thus, it supported calcium preconditioning the PTI machinery for a reinforced response upon subsequent infection. Beyond the PTI, we identified the *RGA4* gene, a member of the NLR (nucleotide-binding leucine-rich repeat) protein family. NLRs function as resistance gene analogs that trigger robust immune responses via direct or indirect recognition of pathogen effectors [39,40], constituting the basis of effector-triggered immunity (ETI)—the second tier of the plant immune system alongside PTI [41]. Our results showed that the *RGA4* gene was already significantly expressed after calcium treatment alone, demonstrating that calcium activates both PTI and ETI. This concurrent engagement of the two major immune branches provides dual-layer assurance for calcium-enhanced disease resistance in barley.

Additionally, secondary metabolic defense pathways were activated (Figure 3 and Figure 4). Among them, flavonoid biosynthesis genes in the phenylpropanoid pathway were significantly enriched. The phenylpropanoid pathway is one of the most important secondary metabolic routes in plants, producing a wide range of defense-related compounds such as lignin, phytoalexins, and flavonoids [42]. Flavonoids, in particular, are well-known for their antimicrobial activity and their roles in reactive oxygen species (ROS) scavenging and signaling during plant–pathogen interactions [43,44]. We also identified multiple transcription factors from the WRKY, MYB, and NAC families that were differentially expressed after calcium treatment. These transcription factor families are among the largest and most studied regulators of plant stress responses [45]. WRKY transcription factors are primarily involved in regulating defense-related gene expression through binding to W-box elements in promoter regions; MYB factors often function in phenylpropanoid metabolism and stress signaling; and NAC proteins are known to integrate developmental and stress signals, particularly during biotic and abiotic stress responses [46]. These transcription factors are known to act as key hubs that link upstream calcium signals to the expression of downstream defense genes, and they may form a regulatory network that coordinates the expression of various defense-related genes [47,48,49].

Our transcriptomic analysis also revealed that exogenous calcium treatment results in significant enrichment of DEGs involved in cutin, suberin, and wax biosynthesis, as well as in linoleic acid metabolism. These pathways are known to contribute to the formation of epidermal protective barriers that impede pathogen invasion, suggesting that calcium treatment primes barley to establish physical defenses prior to infection [50,51]. Among the relevant genes, the *FAR4* gene, which encodes fatty acyl reductase 4, is a key enzyme catalyzing the reduction of fatty acyl-CoA to fatty alcohols [52]. It is a critical step in the biosynthesis of cuticular wax, suberin, and sporopollenin, and *FAR4* was significantly upregulated by calcium treatment alone. However, upon inoculation, this calcium-induced upregulation was altered, with no significant difference observed between calcium-supplemented and control plants. These findings imply that the pathogen may actively suppress the synthesis of physical barriers (cutin/wax), and that calcium treatment is insufficient to counteract this suppression.

Thionins are a class of small, cysteine-rich peptides in plants that exhibit toxicity and antimicrobial properties, showing broad-spectrum cytotoxicity against various organisms and eukaryotic cell lines; they are thought to participate in plant protection against pathogens by directly acting on membranes [53,54]. The *THN3* gene, which encodes a thionin protein, is activated and significantly upregulated after calcium treatment.

In summary, calcium treatment enhances barley’s intrinsic immunity through coordinated activation of immune signaling (PTI/ETI), regulatory networks (WRKY/MYB/NAC), physical barriers (cutin/wax), and antimicrobial peptides (THN3), thereby enabling resistance against *M. oryzae*.

## Figures and Tables

**Table 1:** Statistical results of differential genes.

Compare	All	Up	Down	Threshold
CK vs. Ca50	1121	553	568	DESeq2 padj ≤ 0.05 |log2FoldChange| ≥ 1.0
CK_P vs. Ca50_P	1353	577	776	DESeq2 padj ≤ 0.05 |log2FoldChange| ≥ 1.0
CK vs. CK_P	6542	2835	3707	DESeq2 padj ≤ 0.05 |log2FoldChange| ≥ 1.0
Ca50 vs. Ca50_P	6572	2900	3672	DESeq2 padj ≤ 0.05 |log2FoldChange| ≥ 1.0

**Table 2:** Primers used in the study.

Gene	Primer Name	Sequence	Length
*HvEF1a*	HvEF1a-F	ATGATTCCCACCAAGCCCAT	101 bp
	HvEF1a-R	ACACCAACAGCCACAGTTTGC	
*THN3*	THN3-F	CAGGTCCAGGTGGAGGGA	115 bp
	THN3-R	TACAACCACAAGCACCTGCAC	
*FAR4*	FAR4-F	TGTTCGGGCTTCTAAGGGAG	190 bp
	FAR4-R	ACTCCCATCACGTTCACGTC	
*RGA4*	RGA4-F	AGAGCTGGTGGCGACCATT	110 bp
	RGA4-R	GTTGCTTCTCCAATCCGTCC	

## Data Availability

Restrictions apply to the availability of these data. Data were obtained from National Center for Biotechnology Information (NCBI) and are available [http://www.ncbi.nlm.nih.gov/] with the permission of National Center for Biotechnology Information (NCBI).

## Supplementary Materials

The following supporting information can be downloaded at https://www.mdpi.com/article/10.3390/ijms27156544/s1.

## References

[1] Newton A.C., Flavell A.J., George T.S., Leat P., Mullholland B., Ramsay L., Revoredo-Giha C., Russell J., Steffenson B.J., Swanston J.S. (2011). Crops that feed the world 4. Barley: A resilient crop? Strengths and weaknesses in the context of food security. Food Secur..

[2] Bai Y., Luo X., Qian W., Geng X., Bi X., Zhang Y. (2025). Identification and Analysis of the AP2/ERF Gene Family in Barley Based on Pan-Genome and Pan-Transcriptome. J. Agric. Food Chem..

[3] Hill C.B., Angessa T.T., Zhang X.Q., Chen K., Zhou G., Tan C., Wang P., Westcott S., Li C. (2021). A global barley panel revealing genomic signatures of breeding in modern Australian cultivars. Plant J..

[4] Murray G.M., Brennan J.P. (2010). Estimating disease losses to the Australian barley industry. Australas. Plant Pathol..

[5] Li W., Deng Y., Ning Y., He Z., Wang G.L. (2020). Exploiting Broad-Spectrum Disease Resistance in Crops: From Molecular Dissection to Breeding. Annu. Rev. Plant Biol..

[6] Zellerhoff N., Jarosch B., Groenewald J.Z., Crous P.W., Schaffrath U. (2006). Nonhost resistance of barley is successfully manifested against *Magnaporthe grisea* and a closely related *Pennisetum*-infecting lineage but is overcome by *Magnaporthe oryzae*. Mol. Plant Microbe Interact..

[7] Iqbal O., Yang X., Wang Z., Li D., Wen J., Ding J., Wang C., Li C., Wang Y. (2025). Comparative transcriptome and genome analysis between susceptible Zhefang rice variety Diantun 502 and its resistance variety Diantun 506 upon *Magnaporthe oryzae* infection. BMC Plant Biol..

[8] Aghnoum R., Bvindi C., Menet G., D’hoop B., Maciel J.L.N., Niks R.E. (2019). Host/nonhost status and genetics of resistance in barley against three pathotypes of *Magnaporthe* blast fungi. Euphytica.

[9] Urashima A.S., Alves A.F., Silva F.N., Oliveira D., Gazaffi R. (2017). Host range, mating type and population structure of *Magnaporthe* sp. of a single barley field in São Paulo state, Brazil. J. Phytopathol..

[10] Zhou R.-Q., Jin J.-J., Li Q.-M., Su Z.-Z., Yu X.-J., Tang Y., Luo S.-M., He Y., Li X.-L. (2019). Early Detection of *Magnaporthe oryzae*-Infected Barley Leaves and Lesion Visualization Based on Hyperspectral Imaging. Front. Plant Sci..

[11] Cooper J.G., Donofrio N.M., Caplan J.L., Chaya T.R. (2023). Visualizing Early Infection Sites of Rice Blast Disease (*Magnaporthe oryzae*) on Barley (*Hordeum vulgare*) Using a Basic Microscope and a Smartphone. J. Vis. Exp..

[12] Su’udi M., Kim J., Park J.-M., Bae S.-C., Kim D., Kim Y.-H., Ahn I.-P. (2013). Quantification of Rice Blast Disease Progressions Through Taqman Real-Time PCR. Mol. Biotechnol..

[13] Brabham H.J., Gómez De La Cruz D., Were V., Shimizu M., Saitoh H., Hernández-Pinzón I., Green P., Lorang J., Fujisaki K., Sato K. (2024). Barley MLA3 recognizes the host-specificity effector Pwl2 from *Magnaporthe oryzae*. Plant Cell.

[14] Zellerhoff N., Jansen M., Schaffrath U. (2008). Barley *Rom1* antagonizes *Rar1* function in *Magnaporthe oryzae*-infected leaves by enhancing epidermal and diminishing mesophyll defence. New Phytol..

[15] Ulferts S., Delventhal R., Splivallo R., Karlovsky P., Schaffrath U. (2015). Abscisic acid negatively interferes with basal defence of barley against *Magnaporthe oryzae*. BMC Plant Biol..

[16] Mogga V., Delventhal R., Weidenbach D., Langer S., Bertram P.M., Andresen K., Thines E., Kroj T., Schaffrath U. (2016). *Magnaporthe oryzae* effectors MoHEG13 and MoHEG16 interfere with host infection and MoHEG13 counteracts cell death caused by *Magnaporthe*-NLPs in tobacco. Plant Cell Rep..

[17] Wang J., Diaz J., Hua K., Bellizzi M., Qi L., Zhu L., Qu M., Wang G.-L. (2024). Proteomic identification of apoplastic proteins from rice, wheat, and barley after *Magnaporthe oryzae* infection. Phytopathol. Res..

[18] Liang W., Wang M., Ai X. (2009). The role of calcium in regulating photosynthesis and related physiological indexes of cucumber seedlings under low light intensity and suboptimal temperature stress. Sci. Hortic..

[19] Feng D., Wang X., Gao J., Zhang C., Liu H., Liu P., Sun X. (2023). Exogenous calcium: Its mechanisms and research advances involved in plant stress tolerance. Front. Plant Sci..

[20] Shao H.B., Song W.Y., Chu L.Y. (2008). Advances of calcium signals involved in plant anti-drought. Comptes Rendus Biol..

[21] Bakshi A., Gilroy S. (2024). Calcium signaling in hypoxic response. Plant Physiol..

[22] Kaczmarek M., Fedorowicz-Strońska O., Głowacka K., Waśkiewicz A., Sadowski J. (2016). CaCl_2_ treatment improves drought stress tolerance in barley (*Hordeum vulgare* L.). Acta Physiol. Plant..

[23] Guo T.-r., Chen Y., Zhang Y.-h., Jin Y.-f. (2006). Alleviation of Al Toxicity in Barley by Addition of Calcium. Agric. Sci. China.

[24] Ben Youssef R., Jelali N., Martínez-Andújar C., Abdelly C., Hernández J.A. (2024). Salicylic Acid and Calcium Chloride Seed Priming: A Prominent Frontier in Inducing Mineral Nutrition Balance and Antioxidant System Capacity to Enhance the Tolerance of Barley Plants to Salinity. Plants.

[25] Yan L., Liu S., Li R., Li Z., Piao J., Zhou R. (2024). Calcium enhanced the resistance against *Phoma arachidicola* by improving cell membrane stability and regulating reactive oxygen species metabolism in peanut. BMC Plant Biol..

[26] Xu H., Li C., Wang M., Guo Y., Zhang S., Ge Y. (2024). Enhancement of disease resistance against *Alternaria alternata* in winter jujube fruit by phenyllactic acid through regulating Ca^2+^ signaling transduction and mitogen-activated protein kinase cascades. Postharvest Biol. Technol..

[27] Sun L., Wang Y., Zhang J. (2026). Calcium signaling in plant defense. New Plant Prot..

[28] Kaur A., Bohra Y., Sharma V. (2021). Effect of salicylic acid and calcium chloride in induction of resistance against spot blotch of barley. Plant Dis. Res..

[29] Yan X., Tang B., Ryder L.S., MacLean D., Were V.M., Eseola A.B., Cruz-Mireles N., Ma W., Foster A.J., Osés-Ruiz M. (2023). The transcriptional landscape of plant infection by the rice blast fungus *Magnaporthe oryzae* reveals distinct families of temporally co-regulated and structurally conserved effectors. Plant Cell.

[30] Kawahara Y., Oono Y., Kanamori H., Matsumoto T., Itoh T., Minami E. (2012). Simultaneous RNA-Seq Analysis of a Mixed Transcriptome of Rice and Blast Fungus Interaction. PLoS ONE.

[31] Zhao J., Sun P., Sun Q., Li R., Qin Z., Sha G., Zhou Y., Bi R., Zhang H., Zheng L. (2022). The MoPah1 phosphatidate phosphatase is involved in lipid metabolism, development, and pathogenesis in *Magnaporthe oryzae*. Mol. Plant Pathol..

[32] Zhao J., Chen Y., Ding Z., Zhou Y., Bi R., Qin Z., Yang L., Sun P., Sun Q., Chen G. (2024). Identification of propranolol and derivatives that are chemical inhibitors of phosphatidate phosphatase as potential broad-spectrum fungicides. Plant Commun..

[33] Bellance N., Furt F., Melser S., Lalou C., Thoraval D., Maneta-Peyret L., Lacombe D., Moreau P., Rossignol R. (2020). Doxorubicin Inhibits Phosphatidylserine Decarboxylase and Modifies Mitochondrial Membrane Composition in HeLa Cells. Int. J. Mol. Sci..

[34] Li R., Bi R., Cai H., Zhao J., Sun P., Xu W., Zhou Y., Yang W., Zheng L., Chen X.L. (2023). Melatonin functions as a broad-spectrum antifungal by targeting a conserved pathogen protein kinase. J. Pineal Res..

[35] Kim D., Langmead B., Salzberg S.L. (2015). HISAT: A fast spliced aligner with low memory requirements. Nat. Methods.

[36] Liao Y., Smyth G.K., Shi W. (2014). featureCounts: An efficient general purpose program for assigning sequence reads to genomic features. Bioinformatics.

[37] Livak K.J., Schmittgen T.D. (2001). Analysis of relative gene expression data using real-time quantitative PCR and the 2^−ΔΔCT^ method. Methods.

[38] Jiang Y., Ding P. (2023). Calcium signaling in plant immunity: A spatiotemporally controlled symphony. Trends Plant Sci..

[39] Mierziak J., Wojtasik W. (2024). Epigenetic weapons of plants against fungal pathogens. BMC Plant Biol..

[40] Shepherd S., Yuen E.L.H., Carella P., Bozkurt T.O. (2023). The wheels of destruction: Plant NLR immune receptors are mobile and structurally dynamic disease resistance proteins. Curr. Opin. Plant Biol..

[41] Yu X.Q., Niu H.Q., Liu C., Wang H.L., Yin W., Xia X. (2024). PTI-ETI synergistic signal mechanisms in plant immunity. Plant Biotechnol. J..

[42] Ortiz A., Sansinenea E. (2023). Phenylpropanoid Derivatives and Their Role in Plants’ Health and as antimicrobials. Curr. Microbiol..

[43] Liu W., Feng Y., Yu S., Fan Z., Li X., Li J., Yin H. (2021). The Flavonoid Biosynthesis Network in Plants. Int. J. Mol. Sci..

[44] Shen N., Wang T., Gan Q., Liu S., Wang L., Jin B. (2022). Plant flavonoids: Classification, distribution, biosynthesis, and antioxidant activity. Food Chem..

[45] Reboledo G., Agorio A., Ponce De León I. (2022). Moss transcription factors regulating development and defense responses to stress. J. Exp. Bot..

[46] Meraj T.A., Fu J., Raza M.A., Zhu C., Shen Q., Xu D., Wang Q. (2020). Transcriptional Factors Regulate Plant Stress Responses through Mediating Secondary Metabolism. Genes.

[47] Xiong H., He H., Chang Y., Miao B., Liu Z., Wang Q., Dong F., Xiong L. (2025). Multiple roles of NAC transcription factors in plant development and stress responses. J. Integr. Plant Biol..

[48] Yu Y., Zhang S., Yu Y., Cui N., Yu G., Zhao H., Meng X., Fan H. (2023). The pivotal role of MYB transcription factors in plant disease resistance. Planta.

[49] Pandey S.P., Somssich I.E. (2009). The role of WRKY transcription factors in plant immunity. Plant Physiol..

[50] Ziv C., Zhao Z., Gao Y.G., Xia Y. (2018). Multifunctional Roles of Plant Cuticle During Plant-Pathogen Interactions. Front. Plant Sci..

[51] Pollard M., Beisson F., Li Y., Ohlrogge J.B. (2008). Building lipid barriers: Biosynthesis of cutin and suberin. Trends Plant Sci..

[52] Domergue F., Vishwanath S.J., Joubès J., Ono J., Lee J.A., Bourdon M., Alhattab R., Lowe C., Pascal S., Lessire R. (2010). Three *Arabidopsis* fatty acyl-coenzyme A reductases, FAR1, FAR4, and FAR5, generate primary fatty alcohols associated with suberin deposition. Plant Physiol..

[53] Stec B. (2006). Plant thionins—The structural perspective. Cell. Mol. Life Sci..

[54] Sels J., Mathys J., De Coninck B.M., Cammue B.P., De Bolle M.F. (2008). Plant pathogenesis-related (PR) proteins: A focus on PR peptides. Plant Physiol. Biochem..

